# Supplementary material for: TILLING-by-Sequencing+ to Decipher Oil Biosynthesis Pathway in Soybeans: A New and Effective Platform for High-Throughput Gene Functional Analysis
Source: Int J Mol Sci. 2021 Apr 19;22(8):4219. doi: 10.3390/ijms22084219 (PMC8073088; doi:10.3390/ijms22084219)
Supplement: Supplementary file 1 [file ijms-22-04219-s001.zip › ijms-1172381-supplementary.pdf]

TILLING-by-sequencing+ to decipher oil biosynthesis pathway in soybeans: A new and effective platform for high-throughput gene functional analysis

Naoufal Lakhssassi[1*], Zhou Zhou[1,2*], Mallory A. Cullen[1], Oussama Badad[1], Abdelhalim El Baze[1], Oumaima Chettou[1], Mohamed G. Embaby[3], Dounya Knizia[1], Shiming Liu[1], Leandro G. Neves[2], & Khalid Meksem[1]

1Department of Plant, Soil, and Agricultural Systems, Southern Illinois University, Carbondale, IL 62901, USA.
2Plant Science Department, McGill University, Montreal, QC H9X 3V9, Canada.
3Department of Animal Science, Food, and Nutrition, Southern Illinois University, Carbondale, IL 62901, USA.
4RAPiD Genomics, Gainesville, FL 32601, USA.

*These authors contributed equally to the work.
Corresponding author: meksem@siu.edu.

Supplementary Data

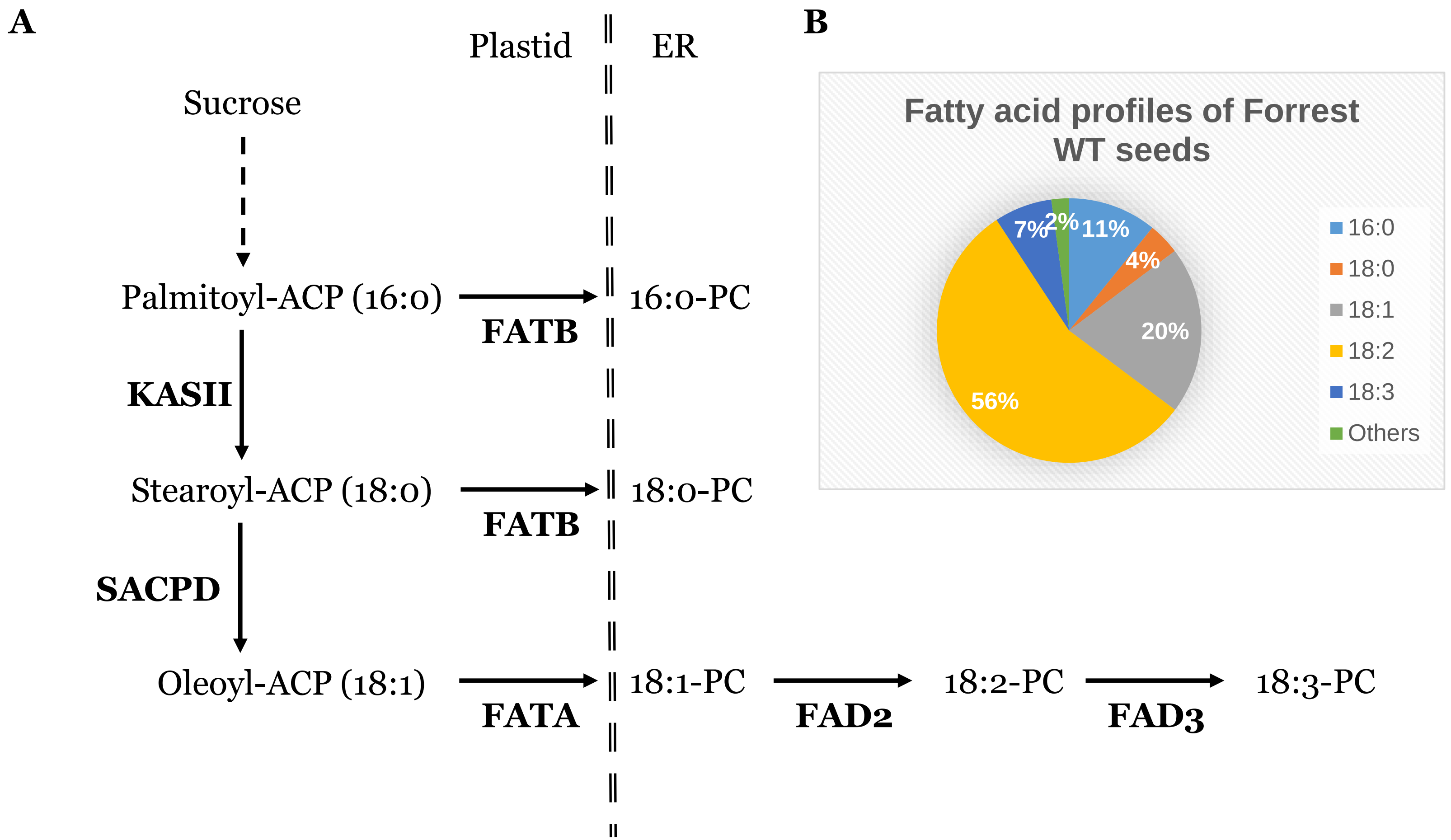

Figure S1. (A) An overview of fatty acid biosynthetic pathway in soybean seed. **(B)** The distribution of fatty acid composition in the Forrest wild type seeds. Abbreviations: enzymatic reactions are in bold: KASII, ketoacyl-ACP synthase II; SACPD, stearoyl-ACP desaturase; FATA, acyl-ACP thioesterase A; FATB, acyl-ACP thioesterase B; FAD2, omega-6 fatty acid desaturase 2; FAD3, omega-3 fatty acid desaturase. ER, endoplasmic reticulum; ACP, acyl carrier protein; PC, phosphatidylcholine.

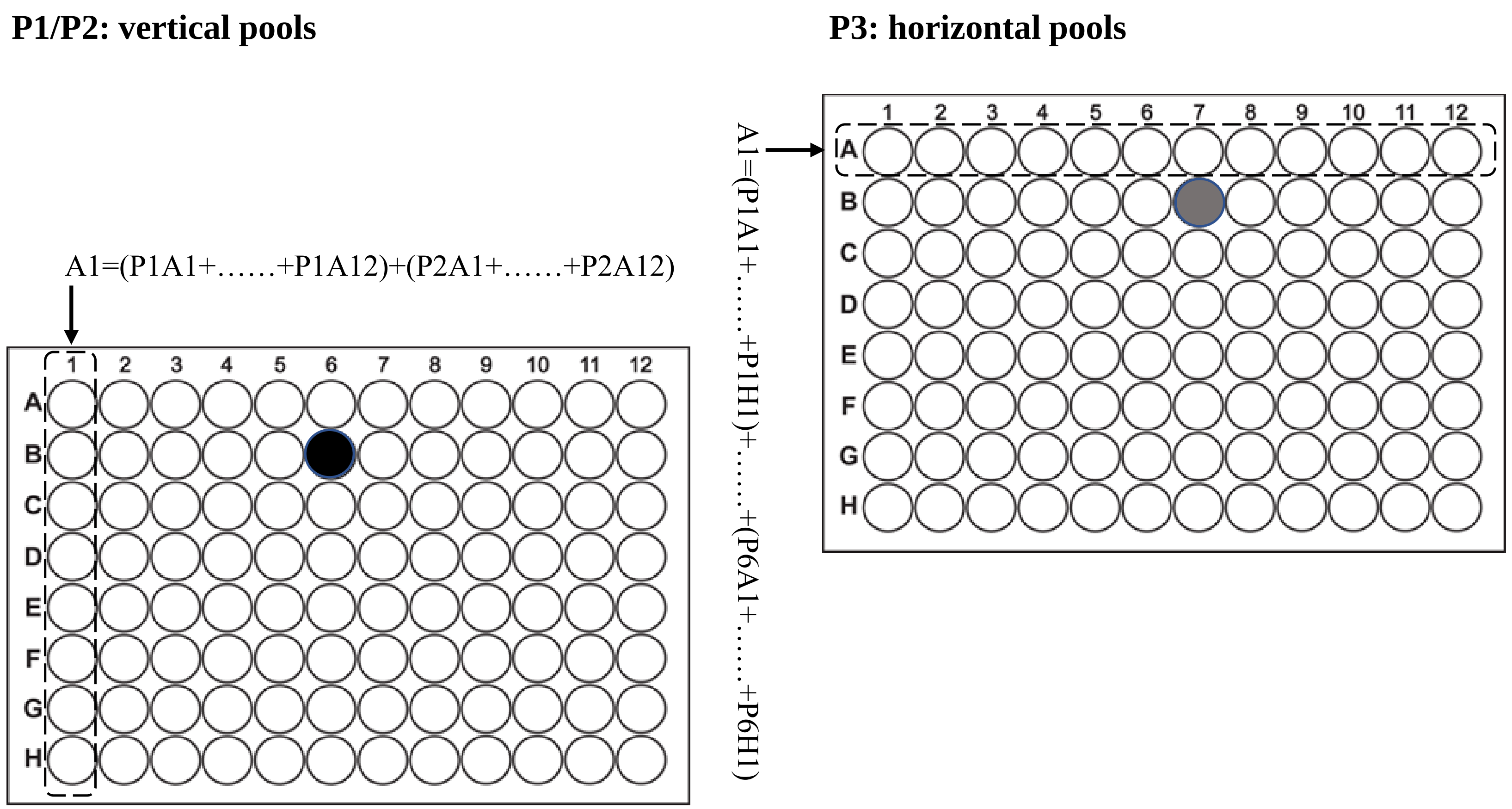

Figure S2. Pooling and demultiplexing in soybean mutant library. 168 vertical pools, 24 DNA samples in each pool, were included in P1 and P2 while 84 horizontal pools were in P3 with 48 DNA samples in each pool. Two candidate *GmSACPD-C* mutant lines were determined from analyzing the same mutation appearing in P1B6 (Black circle) and in P3B7 (Grey circle), for example.

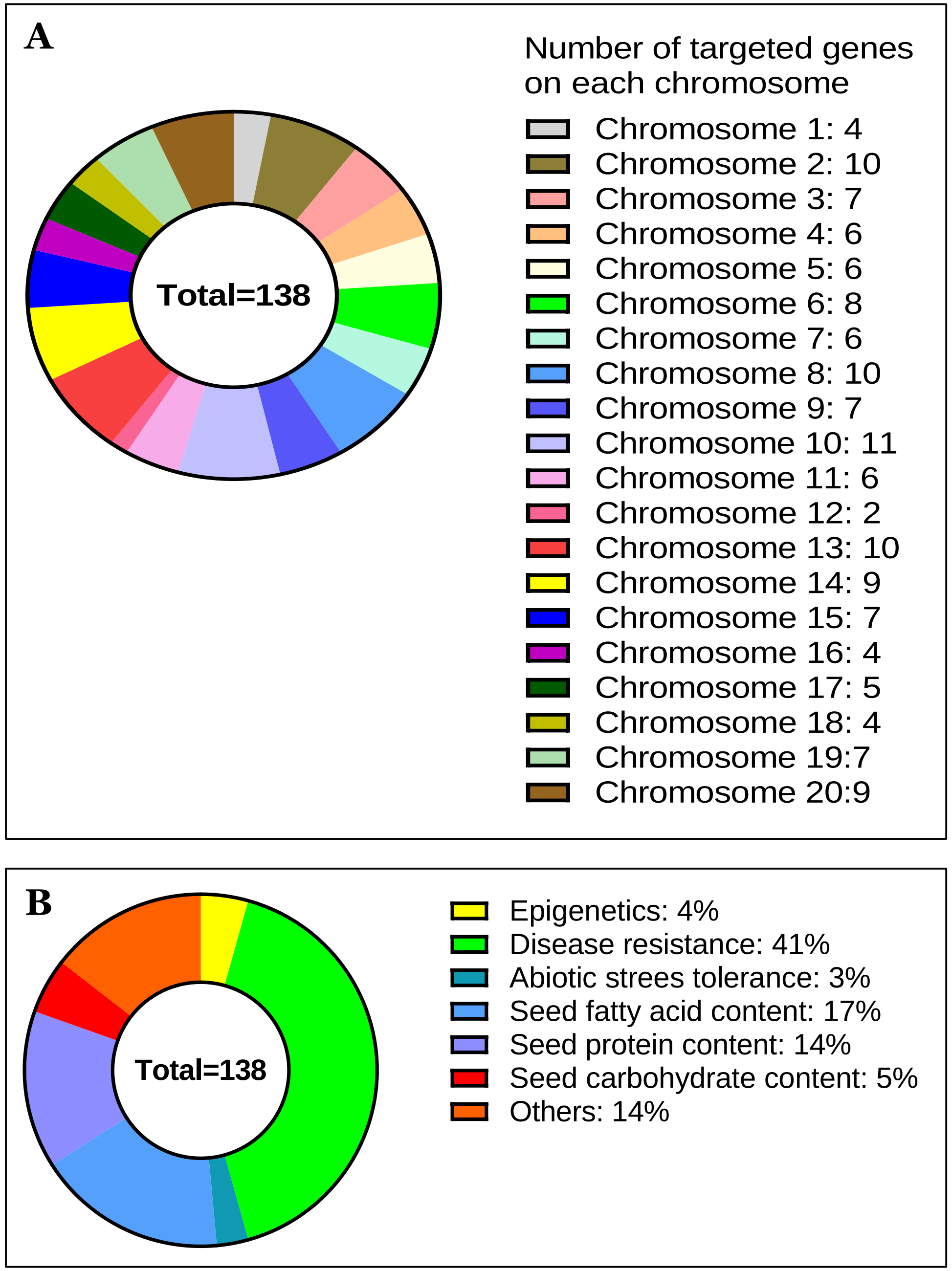

Figure S3. The distribution of soybean genes screened by TbyTCS. **(A)** The number of genes targeted on 20 soybean chromosomes. **(B)** The percentage of genes in categories divided by traits.

Table S1. Information of probes designed for six fatty acid desaturase genes in TILLING-by-Sequencing[+].

Gene ID	Chromosome No.	start position	stop position	Glyma No.	probes	bp covered	bp in region	coverage
GmSACPD-C	Chr14	2E+07	2E+07	Glyma.14G121400	20	880	910	0.97
	Chr14	2E+07	2E+07	Glyma.14G121400	21	920	938	0.98
GmFAD2-1A	Chr10	5E+07	5E+07	Glyma.10G278000	12	560	562	1
	Chr10	5E+07	5E+07	Glyma.10G278000	32	1360	1366	1
GmFAD2-1B	Chr20	4E+07	4E+07	Glyma.20G111000	26	1120	1145	0.98
	Chr20	4E+07	4E+07	Glyma.20G111000	19	840	877	0.96
	Chr20	4E+07	4E+07	Glyma.20G111000	32	1360	1381	0.98
GmFAD3A	Chr14	5E+07	5E+07	Glyma.14G194300	8	400	406	0.99
	Chr14	5E+07	5E+07	Glyma.14G194300	7	360	377	0.95
	Chr14	5E+07	5E+07	Glyma.14G194300	4	240	343	0.7
	Chr14	5E+07	5E+07	Glyma.14G194300	15	760	829	0.92
	Chr14	5E+07	5E+07	Glyma.14G194300	6	320	329	0.97
	Chr14	5E+07	5E+07	Glyma.14G194300	8	400	429	0.93
GmFAD3B	Chr02	4E+07	4E+07	Glyma.02G227200	7	360	365	0.99
	Chr02	4E+07	4E+07	Glyma.02G227200	7	360	377	0.95
	Chr02	4E+07	4E+07	Glyma.02G227200	23	1000	1021	0.98
	Chr02	4E+07	4E+07	Glyma.02G227200	6	320	329	0.97
	Chr02	4E+07	4E+07	Glyma.02G227200	9	440	451	0.98
GmFAD3C	Chr18	6E+06	6E+06	Glyma.18G062000	7	360	389	0.93
	Chr18	6E+06	6E+06	Glyma.18G062000	32	1360	1389	0.98
	Chr18	6E+06	6E+06	Glyma.18G062000	7	360	377	0.95
	Chr18	6E+06	6E+06	Glyma.18G062000	7	360	399	0.9

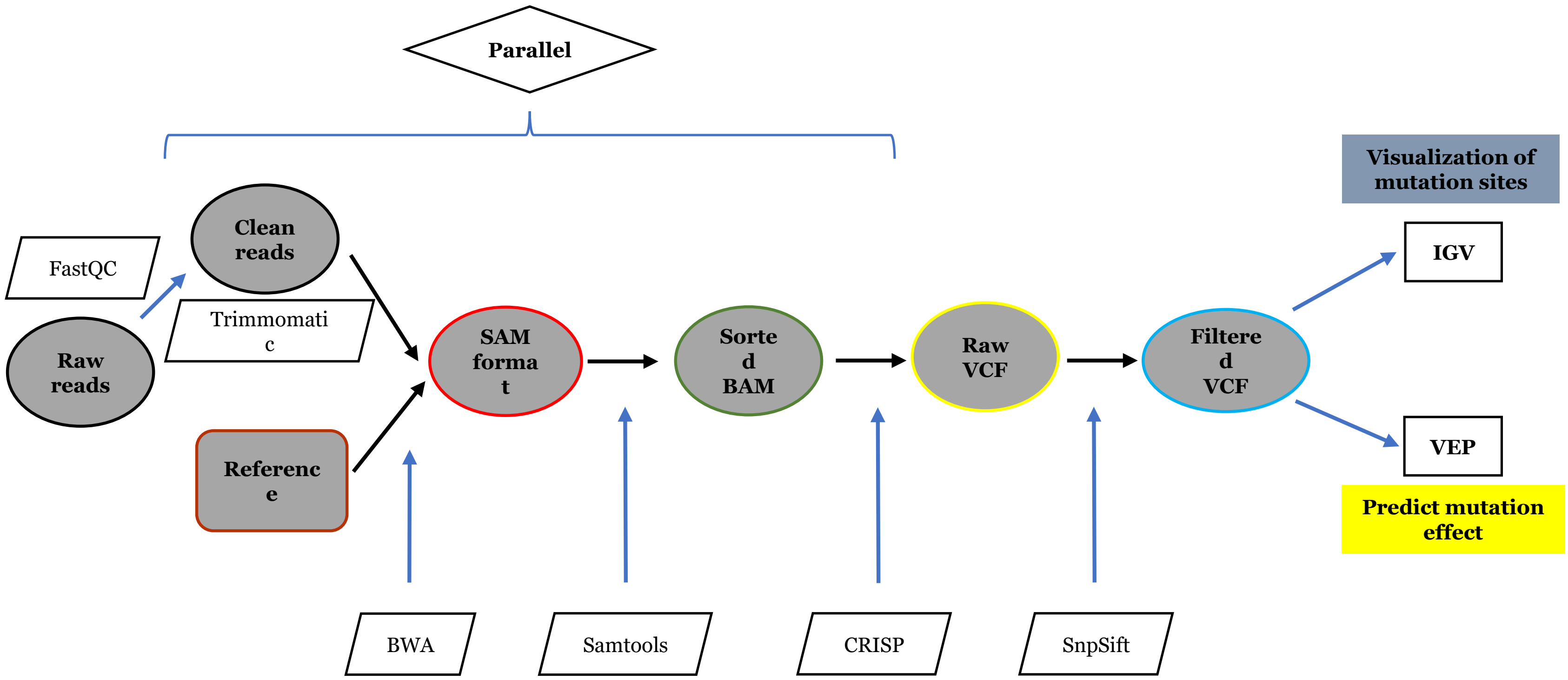

Figure S4. An overview of the SNP calling pipeline. The generated raw sequencing reads were quality checked by FastQC and then processed with parallel using an automatically shell program consisted of bwa and samtools. The sorted bam files were used for SNP calling by CRISP to generate VCF files. The raw VCF files were finally filtered by SnpSift. Using Integrative Genomics Viewer (IGV), mutant lines were recovered from the filtered VCF files. The mutation effect was predicted by Variant Effect Predictor (VEP) program at Ensembl Plants release 45.

Table S2. A summary of EMS induced mutations identified in 138 genes and 6 fatty acid desaturase genes.

Mutations characteristics	138 genes	6 fatty acid biosynthetic genes
**Amplicon size (bp)**	358152	14440
**Base changes**	6362	274
**G > A**	2698	111
**C > T**	2550	107
**Others**	1114	56
**Mutation density (kb)**	1/227	1/212
**Amino acid substitutions**	3785	147
**Missense mutations**	2479	92
**Nonsense mutations**	156	5
**Silent mutations**	1150	50

Table S3. A summary of putative EMS induced mutations in *GmSACPD-C/D* and *GmFAD2-1A*.

Gene ID	Mutant ID	Vertical pool	Horizontal pool	Amplicon size (bp)	Position (bp)	Allele frequency (%)	Nucleotide change	Amino acid substitution
GmSACPD-C (Glyma.14g121400)	F813	P1E5	P3B6	920	17501614	0.017	C305T	P102L
	F714	P1B4	P3B6	920	17501690	0.029	G229A	D77N
	F620	P1D4	P3B6	920	17501684	0.043	C235T	L79F
	F869	P1E5	P3B1	920	17501579	0.017	G340A	E114K
GmSACPD-D (Glyma.13g038600)	F180	P1D1	P3A11	800	11958789	0.017	T335A	V112E
	F425	P1A3	P3A6	720	11957254	0.016	G706A	G236R
GmFAD2-1A (Glyma.10g278000)	F1235	P1C6	P3B11	1360	50014391	0.018	C301T	L101F
	F1284	P1D7	P3C5	1360	50014941	0.225	C851T	P284L
	F1274	P1B7	P3C4	1360	50014835	0.023	C745T	L249=

Table S4. A summary of mutants in *GmSACPD-C* and *GmFAD2-1A/B* genes identified by TILLING-by-Sequencing[+]

Gene ID	Mutant #	Plant ID	Nucleotide change	Amino acid substitution
GmSACPD-C (Glyma.14g121400)	1	F1052	G143A	S48N
	2	F2146	C322T	R108W
	3	F186	G554A	G185E
	4	F1503	C713T	A238V
	5	F1202	G730A	G244R
	6	F1320	G782A	G261D
GmFAD2-1A (Glyma.10g278000)	1	F1356	C88T	P30S
	2	F765	G116A	G39D
	3	F1297	C298T	L100F
	4	F258	A502C	K168Q
	5	F101	G673A	E225K
	6	F1303	C1060T	P354S
GmFAD2-1B (Glyma.20g111000)	1	F966	A338C	H113P
	2	F760	G440A	R147H
	3	F782	G505A	V169I
	4	F215	C695T	S232F
	5	F720	C784T	P262S
	6	F36	C845T	A282V
	7	F913	G984A	M328I
	8	F532	C994T	H332Y
	9	F903	G1129A	E377K
	10	F1792	G1146A	W382*

Table S4 (Continued). A summary of mutants in *GmFAD3* genes identified by TbyTCS.

Gene ID	Mutant #	Plant ID	Nucleotide change	Amino acid substitution
GmFAD3-A (Glyma.14g194300)	1	F1022	C82T	P28S
	2	F1178	C511T	P171S
	3	F180	G830A	G277D
	4	F1012	C835T	L279F
	5	F1033	G1054A	D352N
	6	F1428	G1078A	D360N
GmFAD3-B (Glyma.02g227200)	1	F1173	C94T	P32S
	2	F475	C461T	P154L
	3	F560	G741A	W247*
	4	F728	G845A	G282D
	5	F461	C916T	H306Y
	6	F577	G967A	A323T
GmFAD3-C (Glyma.18g062000)	1	F953	G112A	A38T
	2	F847	G335A	S112N
	3	F846	G383A	G128E
	4	F1012	G800A	W267*
	5	P239	G854A	R285H
	6	F1739	A998C	Q333P

Table S5. The list of soybean fatty acid desaturase genes with their corresponding gene ID, nucleotide sequence characteristics, and protein sequence properties.

Gene name	Gene ID	CDS (bp)	Exons	Protein sequence (aa)	Mol.Wt. (kDa)	pI
GmSACPD-A	Glyma.07G207200	1209	3	402	46.1	6.05
GmSACPD-B	Glyma.02G138100	1176	3	391	44.9	5.94
GmSACPD-C	Glyma.14G121400	1014	2	337	38.6	5.66
GmSACPD-D	Glyma.13G038600	1137	2	378	43.0	7.68
GmFAD2-1A	Glyma.10G278000	1164	2	387	44.7	9.01
GmFAD2-1B	Glyma.20G111000	1164	2	387	44.6	9.00
GmFAD2-2A	Glyma.19G147300	651	2	216	25.5	8.35
GmFAD2-2B	Glyma.19G147400	1152	1	383	43.8	8.70
GmFAD2-2C	Glyma.03G144500	1152	1	383	44.1	8.58
GmFAD2-2D	Glyma.09G111900	1161	1	386	44.2	9.08
GmFAD2-2E	Glyma.15G195200	882	1	293	33.7	7.39
GmFAD3A	Glyma.14G194300	1131	8	376	43.9	8.74
GmFAD3B	Glyma.02G227200	1143	8	380	44.1	8.50
GmFAD3C	Glyma.18G062000	1143	8	380	43.9	8.88
GmFAD3D	Glyma.11G174100	1146	8	381	44.1	8.72
GmFAD7-1	Glyma.18G202600	1362	8	453	51.2	8.45
GmFAD7-2	Glyma.07G151300	1362	8	453	51.2	8.17
GmFAD8-1	Glyma.01G120400	1359	8	452	51.4	7.40
GmFAD8-2	Glyma.03G056700	1362	8	453	51.3	7.39

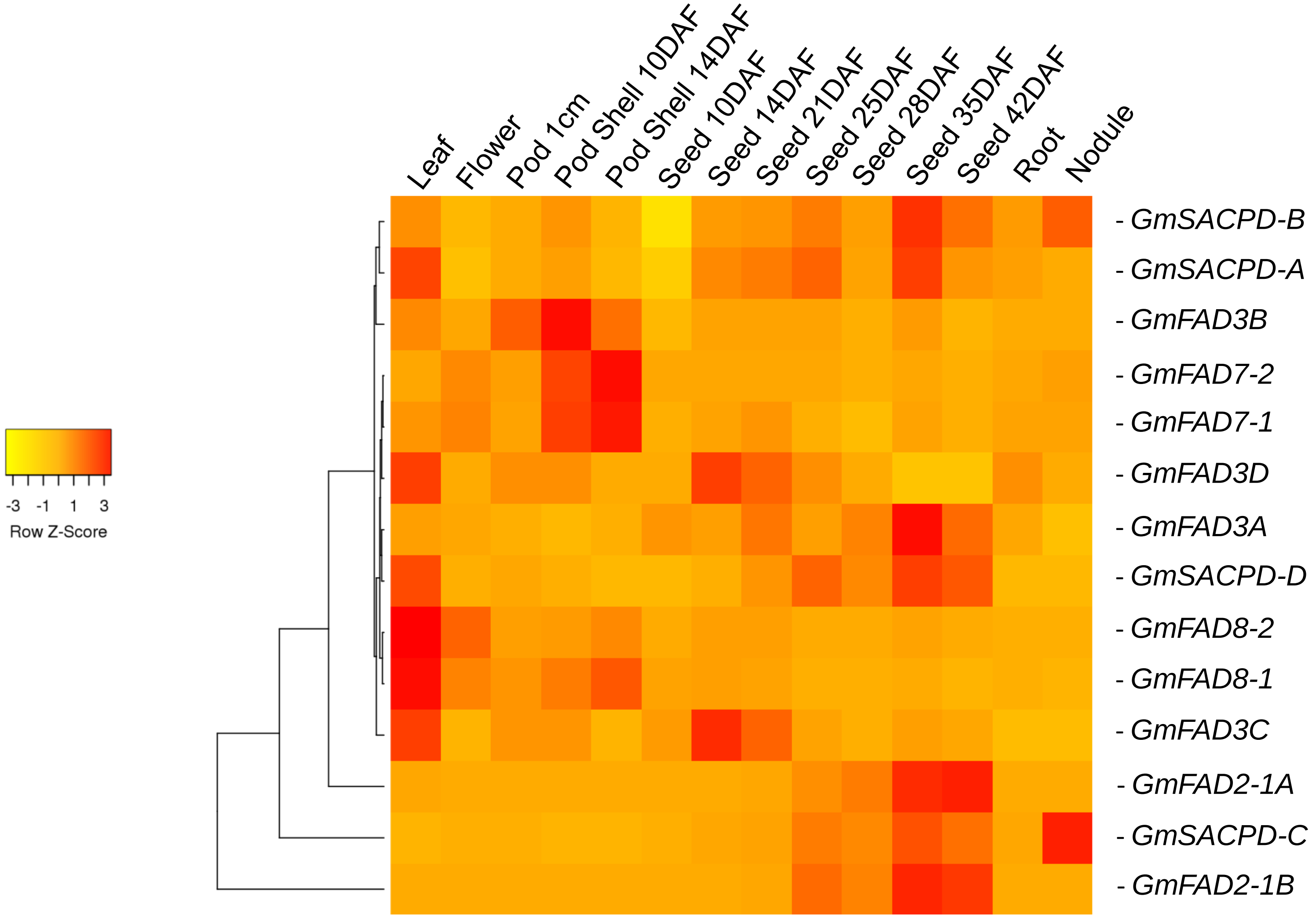

Figure S5. Heatmap of tissue-specific expression profiles of soybean fatty acid desaturases genes. RNAseq expression data were collected from soybase. Color key represents the relative transcript abundance from low (yellow) to high (Red).

Table S6. Divergence and duplication of fatty acid desaturases gene pairs in soybean.

Fatty acid desaturase gene pairs	Ka	Ks	Ka/Ks	Duplication time (Mya)	Duplication type
GmSACPD-A-GmSACPD-B	0.01	0.20	0.050	16.39	Segmental
GmSACPD-C-GmSACPD-D	0.09	0.44	0.205	36.07	Segmental
GmFAD2-1A-GmFAD2-1B	0.03	0.13	0.231	10.66	Segmental
GmFAD2-2A-GmFAD2-2B	0.08	0.31	0.251	25.41	Tandem
GmFAD2-2C-GmFAD2-2D	0.13	1.30	0.100	106.56	Segmental
GmFAD3A-GmFAD3B	0.02	0.13	0.154	10.66	Segmental
GmFAD3C-GmFAD3D	0.02	0.12	0.167	9.84	Segmental
GmFAD7-1-GmFAD7-2	0.03	0.09	0.333	7.38	Segmental
GmFAD8-1-GmFAD8-2	0.02	0.14	0.143	11.48	Segmental

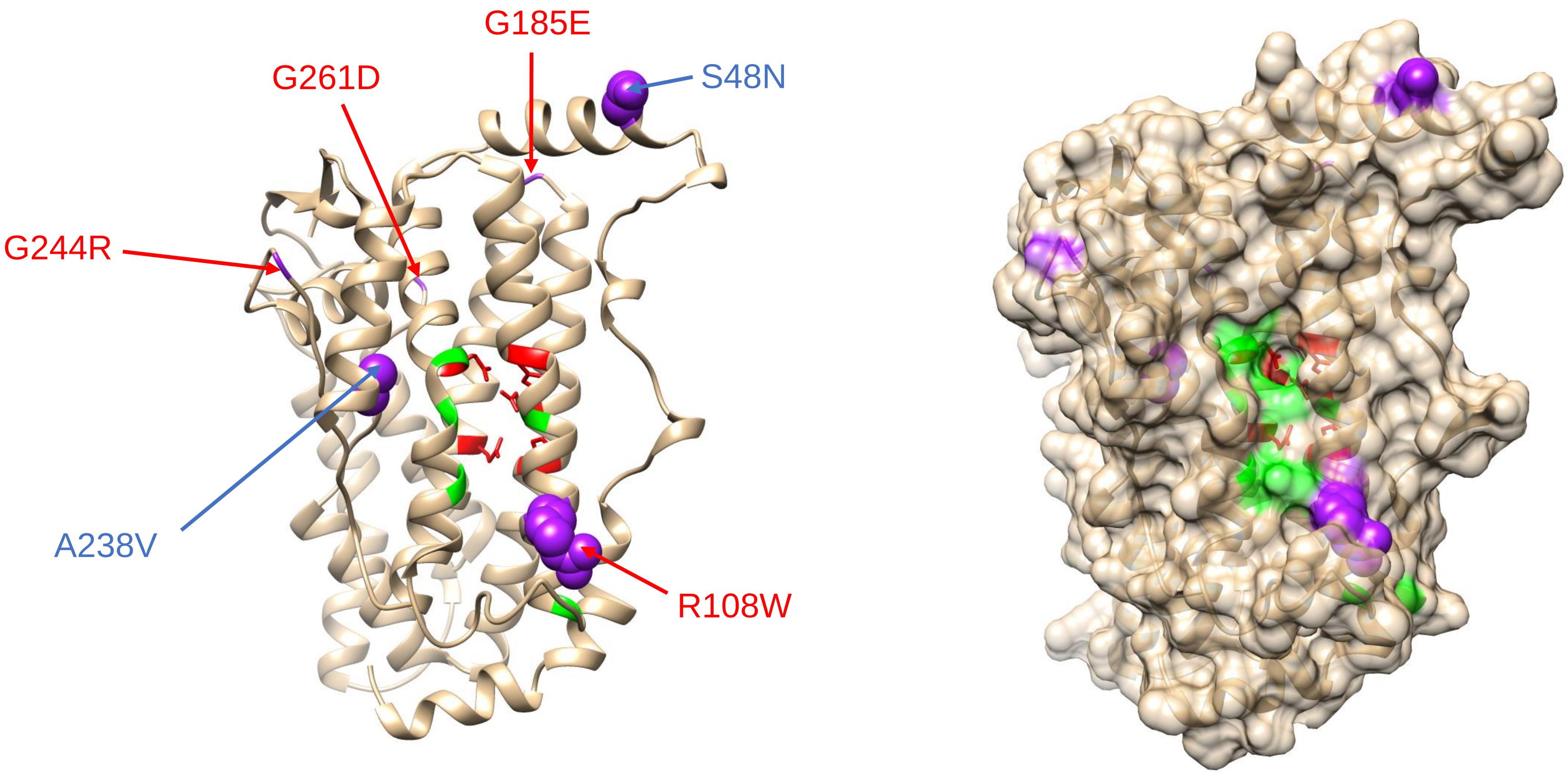

Figure S6. Structural analysis of six *GmSACPD-C* mutants in homology modeling of cv Forrest GmSACPD-C with catalytic residues mapped. Six *GmSACPD-C* mutations are highlighted in purple, from which four missense mutations conferred high stearic acid content in soybean seed are marked in red. An di-iron center is shown in different colors.
